# Immunolocalization and Expression of JAK1 and JAK3 in the Skin of Dust Mite-Sensitive Beagle Dogs before and after Allergen Exposure

**DOI:** 10.3390/vetsci10080512

**Published:** 2023-08-08

**Authors:** Roberta Sartori, Kim Ahrens, Rachel Wilkes, Rosanna Marsella

**Affiliations:** 1 Servizi Dermatologici Veterinari, 20125 Milano, Italy; sartori.roberta@hotmail.com; 2 Department of Small Animal Clinical Science, College of Veterinary Medicine, University of Florida, Gainesville, FL 32608, USA; sciencematters2me@gmail.com (K.A.); rachelsusansanford@gmail.com (R.W.)

**Keywords:** Janus kinase pathway, JAK1, JAK3, canine atopic dermatitis

## Abstract

**Simple Summary:**

Our study aimed to stain a cellular pathway that is a potential target of drugs to treat allergies. In our study, we took skin samples from dogs allergic to dust, before and after exposure to dust mites, and stained those samples for this pathway. We found that this pathway was present in skin cells and that exposure to the dust mites changed how this pathway was expressed. These results confirm that this pathway plays a role in the course of allergic responses and that topical creams targeting this pathway can be promising to treat allergic skin disease.

**Abstract:**

Janus kinase (JAK) pathways have emerged as targets of treatment, yet localization and expression of JAK1 and JAK3 in canine atopic skin have not been studied. This study aimed to compare the localization and expression of JAK1 and JAK3 in the skin of atopic dogs before and after allergen exposure. Skin biopsies taken from atopic beagles sensitized to house dust mites (HDM) before (D0) and after four weeks (D28) of allergen exposure were stained. Staining was subjectively scored by examiners unaware of the source of the slides. Image J was used for the semiquantitative assessment of staining intensity. JAK1 and JAK3 staining was epidermal and dermal. JAK1 staining was cytoplasmic, primarily found in basal keratinocytes and dermal cells, while JAK 3 was nuclear (all epidermal levels and on dermal inflammatory cells). Epidermal thickness was significantly higher on D28 than on D0 (*p* < 0.0001). For JAK1, epidermal staining divided by epithelial thickness was significantly lower on D28 (*p* = 0.0002) compared to D0. For JAK3 staining, intensity in the dermis was significantly higher on D28 (*p* = 0.0405) compared to D0. We conclude that decreased expression of JAK1 in the epidermis and increased expression of JAK3 in the dermis of atopic dogs occur after allergen exposure.

## 1. Introduction

Janus kinase (JAK) enzymes are involved in intracellular signaling pathways activated by cytokines dysregulated in various conditions ranging from cancer to a variety of inflammatory diseases such as allergies [1]. The JAK pathway has been identified in recent years as an important target for the treatment of both inflammatory and autoimmune diseases in human medicine. In veterinary medicine, the main experience with JAK inhibitors has been with oclacitinib, which received FDA approval for the management of canine allergic skin diseases in 2013. Oclacitinib has been very successful in providing fast relief from pruritus and a high level of efficacy to decrease the severity of signs associated with canine atopic dermatitis [2]. Oclacitinib is considered a selective JAK 1 inhibitor at labeled doses, thus primarily interfering with the signaling of allergic cytokines and minimally interfering with hematopoiesis and natural immunity. This is an important distinction between oclacitinib and many JAK inhibitors approved for use in human medicine that target other JAK pathways. Interestingly, despite the extensive use of oclacitinib in the past decade, no study has reported on the expression of JAKs in canine skin. Currently, the localization and expression of JAK1 and JAK3 in canine skin have never been described, and the effect of allergen exposure on the expression of JAKs in canine skin is unknown.

In normal human epidermis, JAK expression and distribution have been characterized by Nishio et al. [3]. In that study, a strong immunoreactivity for JAK1 and JAK3 was described in the skin, and differential expression of various JAKS was reported in the epidermis, highlighting a likely role for JAKS in keratinocyte differentiation. A few years later, immunohistochemistry on healthy and lesional human skin was performed by Juczynska et al., showing the same staining patterns [4]. JAK1 was reported to be cytoplasmic, and JAK3 was found to be perinuclear and nuclear in both studies [3,4]. JAK 1 was found throughout the epidermis, and the horny cell layer was not stained [3]. The expression of JAK3 was found throughout the epidermis with the horny and granular layers being strongly stained by the antibody [4].

The expression of the JAK-STAT pathway in the epidermis suggests its role in keratinocyte differentiation, and this was nicely demonstrated in a Japanese study in 2015 [5]. In that study, the role of JAK-STAT signaling in skin barrier function was evaluated from the perspective of atopic dermatitis. Microarray analysis with a human skin equivalent model revealed that IL-4/IL-13 dominantly affected keratinocyte differentiation, presumably through JAK-STAT signaling. It was also found that the JAK inhibitor JTE-052 promoted filaggrin and loricrin protein production and that STAT3 is the key transcriptional factor in keratinocyte differentiation. These findings supported the notion that JAK-STAT3 signaling plays a key role in modulating keratinocyte differentiation [5]. There is also evidence that the JAK/STAT pathway is essential to the normal functioning of the immune system [6]. Stimulation of the JAK/STAT pathway facilitates intercellular communication and plays a significant role in cell processes such as proliferation, growth, differentiation, migration, and apoptosis [7].

The objectives of our study were to compare localization and expression of JAK1 and JAK3 in the skin of dust mite-allergic atopic dogs before and after epicutaneous allergen exposure. Since patients with naturally occurring disease can have a combination of immunologically acute and chronic lesions, the availability of a research model in which the lesions can be triggered by allergen exposure and monitored over time allowing sequential biopsies is of great benefit. A colony of atopic beagles that naturally develop atopic dermatitis and develop clinical flares after exposure to dust mites has been validated as a model for canine atopic dermatitis [8,9,10]. Dogs belonging to this colony were used for this study, and sequential allergen challenges were conducted over the course of 28 days [8,9,10]. Thus, the aim of our study was to investigate the localization of JAKs in canine atopic skin before (Day 0) and after allergen exposure (Day 28) to see if changes in the expression and distribution of JAKs occur. The interest to look at changes in the course of allergen exposure was motivated by the fact that JAK1 and JAK3 are modulated by signaling molecules that play an important role in modulating pruritus and inflammation [2,11,12]. JAK/STAT signaling is inhibited by suppressors of cytokine signaling proteins (SOCS) [13]. The SOCS proteins regulate the balance between T helper cells with SOCS3 expressed in Th2 cells and atopic patients [13]. Therefore, the hypothesis of our study was that JAK expression in lesional atopic skin after allergen exposure is decreased compared to non-lesional atopic skin. Documentation of the expression of JAKs in canine skin is important to build the foundation for the pursuit of topical JAK inhibitors in veterinary medicine. The modulation of JAK expression upon allergen exposure is of particular interest for future therapeutic purposes.

## 2. Materials and Methods

All procedures were approved by the Institutional Animal Care and Use Committee. No client consent was obtained in this study as the dogs were research dogs and not privately-owned pets.

### 2.1. Animals

Atopic beagles (atopic dogs n = 8). Eight atopic beagles, epicutaneously sensitized to Dermatophagoides farinae at a young age and known to develop atopic dermatitis flares upon environmental exposure to the allergen were used [8,9,10].

### 2.2. Allergen Challenges

All beagles were epicutaneously challenged with house dust mites prepared from crude Dermatophagoides farinae (Greer; Lenoir, NC, USA) mixed with phosphate-buffered saline (PBS) [9,10]. Allergen solution was applied to the inguinal and chest area twice a week for four weeks [9,10]. Doses and frequency of administration of the allergen were based on prior studies [9,10]. No tape stripping was undertaken prior to application of the allergen. The allergen was simply massaged on the skin, and no method of occlusion was used.

### 2.3. Biopsies

Skin biopsies from the inguinal or chest area were taken at baseline (Day 0) and after four weeks (Day 28) of allergen exposure. Baseline biopsies were from non-lesional skin while biopsies on Day 28 were from lesional areas. Lesions after allergen exposure primarily consisted of erythematous macules and papules.

### 2.4. Immunohistochemistry

Immunohistochemistry was performed on biopsies as previously described, using mouse monoclonal antibodies against JAK1 (dilution 1:50) and JAK3 (dilution 1:100) (Santa-Cruz Biotechnologies; Dallas, TX, USA). Homologies between canine and human JAKs were confirmed via BLAST. These antibodies detected both phosphorylated and non-phosphorylated forms of the molecules. Negative and positive controls were dog skin stained with mouse IgG, normal human skin as described by Nishio et al. [3], and canine cutaneous T-cell lymphoma, respectively. The latter control was used because neoplastic cells have an aberrant activation of the JAK/STAT pathway, which predisposes to malignancy due to deregulation of proliferation, differentiation, or apoptosis [14].

### 2.5. Evaluation of Immunohistochemical Staining

Five representative fields from each section were examined at ×400 magnification, and digital images were collected using MagnaFire Software v070121-01A (Optronics; Goleta, CA, USA), and blindly scored according to the amount of staining present in the epidermis and dermis subjectively. These images were assessed by four investigators, who scored the intensity of the staining in the epidermis and dermis of each picture on a scale of 0–3 (0 = absent, 3 = severe). The thickness of the epidermis and extent of immunostaining in the epidermis and dermis were evaluated using a subjective scale of 0–4 (0 = baseline, 4 = severe). The scores of the extent and intensity of staining were added, and the mean of the total scores was calculated and used for analysis. Photographs were also used to calculate objectively the amount of immunostaining present in each section semi-quantitatively. For JAK1, each photograph was opened in ImageJ and a pixel intensity threshold was determined to include only those image pixels in immunopositive areas. The epidermis was traced, and the total pixel count of immunopositive per area was calculated [9,10]. For JAK3, an actual count of the number of cells with nuclear staining was opened in image J and counted individually. The number of immunostained cells was calculated by dividing the count of the immunopositive areas by the area of the epidermis [9,10].

### 2.6. Evaluation of Severity of Dermatitis

Severity of dermatitis was evaluated at each time point (Day 0 and Day 28) using a previously validated scoring system (canine atopic dermatitis extent and severity index or CADESI-03) [15]. All evaluations were completed by the same investigator. According to the scoring system used, a score is assigned to various body locations and the total CADESI is the sum of the scores for each body area.

### 2.7. Statistics

Comparisons between days were performed (paired two-tailed *t*-test). Correlations between the severity of dermatitis (CADES) and the percentage of staining for both JAK1 and JAK 3 were evaluated. Data were assessed for normality (Shapiro–Wilk test) and, if found to be normally distributed, correlations were assessed using Pearson’s correlation coefficient, otherwise using Spearman’s correlation coefficient. Statistical analyses were performed using GraphPad Prism v7.00 for Windows (GraphPad Software^®^). A *p* < 0.05 was considered significant.

## 3. Results

### 3.1. Subjective Evaluation

**Figure 1:**
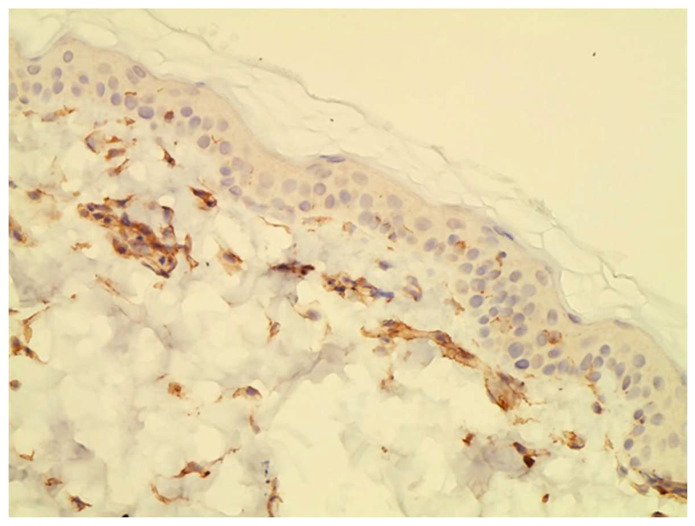
Example of JAK1 staining visible in the cytoplasm of basal keratinocytes and in dermal cells.

**Figure 2:**
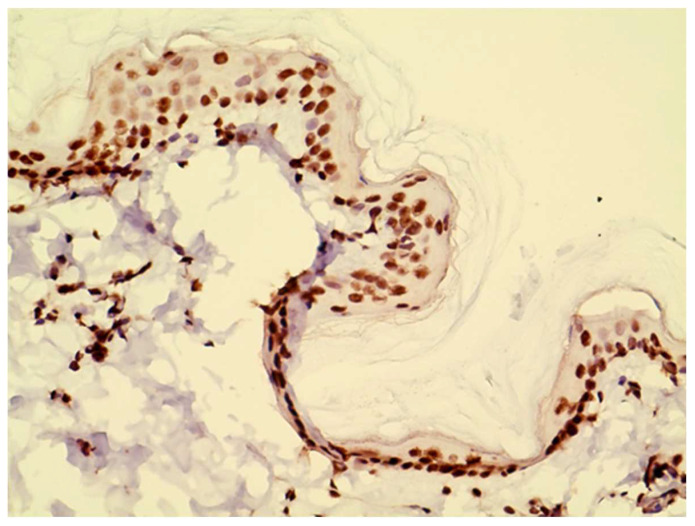
Example of JAK3 staining, which was nuclear at all levels of the epidermis and on inflammatory cells in the dermis.

**Figure 3:**
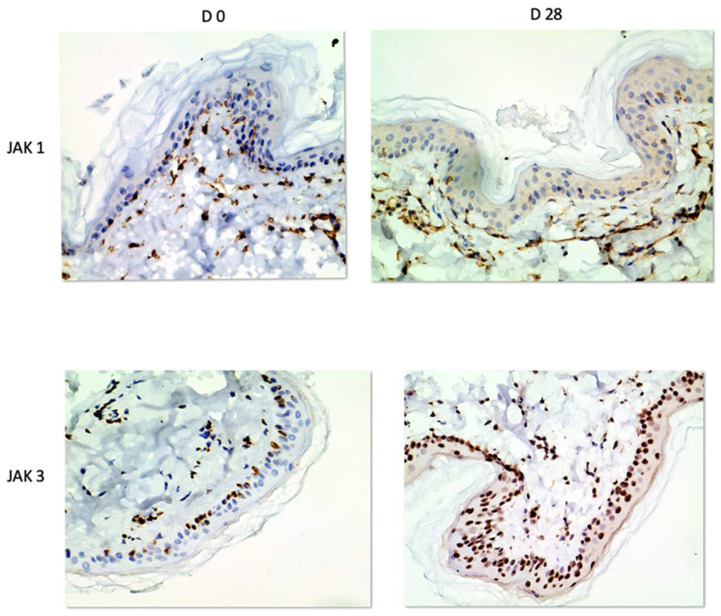
Representative images of biopsies taken at baseline (Day 0, **left images**) and after 28 days of allergen exposure (Day 28, **right images**). Note the staining both in the epidermis and in the dermis.

JAK1 and JAK3 staining was detected in both epidermis and dermis. JAK1 staining was cytoplasmic and primarily seen in basal keratinocytes and on dendritic cells (Figure 1). JAK3 was nuclear at all levels of the epidermis and on inflammatory cells in the dermis. (Figure 2 and Figure 3).

**Figure 4:**
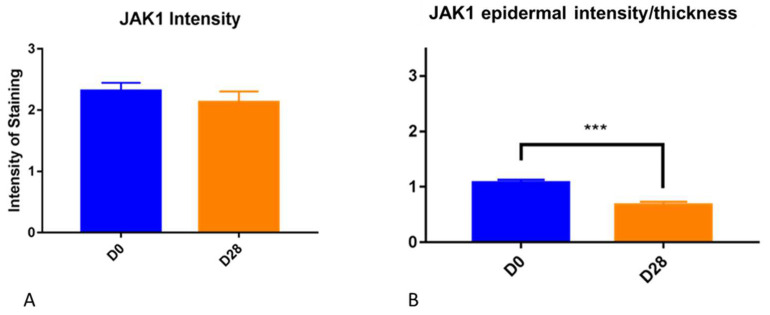
(**A**) JAK1 subjective score for intensity overall was not significantly different between Day 0 and Day 28. (**B**) Epidermal staining divided by epidermal thickness was significantly (***) lower on Day 28 (*p* = 0.0002) compared to Day 0.

Epidermal thickness was significantly higher on Day 28 than on Day 0 (*p* < 0.0001). For JAK1, epidermal staining divided by epidermal thickness was significantly lower on Day 28 (*p* = 0.0002) subjectively (Figure 4). No significant changes were seen in the dermis.

**Figure 5:**
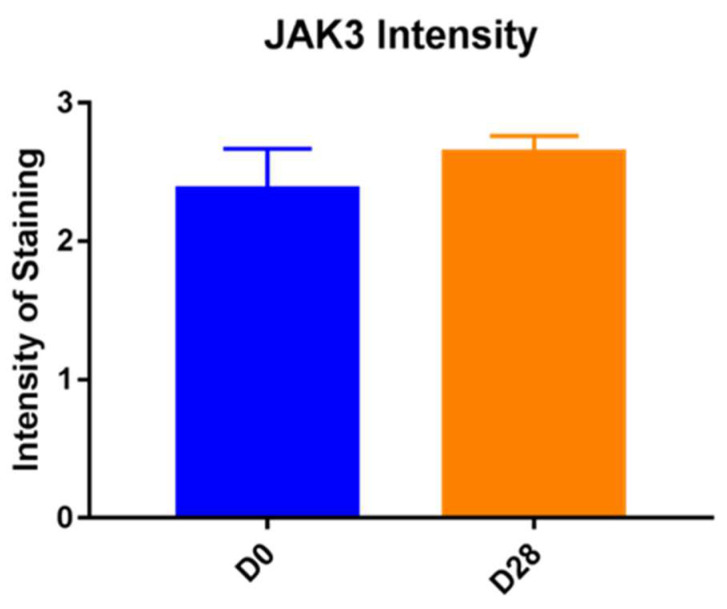
JAK3 intensity of staining is significantly higher in the epidermis on Day 28 compared to Day 0 (*p* = 0.0022).

For JAK3, subjective scores of intensity of staining were significantly higher in the epidermis on Day 28 compared to Day 0 (*p* = 0.0022). For JAK 3 staining, patchiness was detected on average in 20% of pictures scored on Day 0 and 15% on Day 28. Due to variability, this difference was not statistically significant. Cell numbers staining positively for JAK3 were significantly higher in the epidermis on Day 28 (*p* = 0.0012, Figure 5).

### 3.2. Semiquantitative Assessment (Image J)

**Figure 6:**
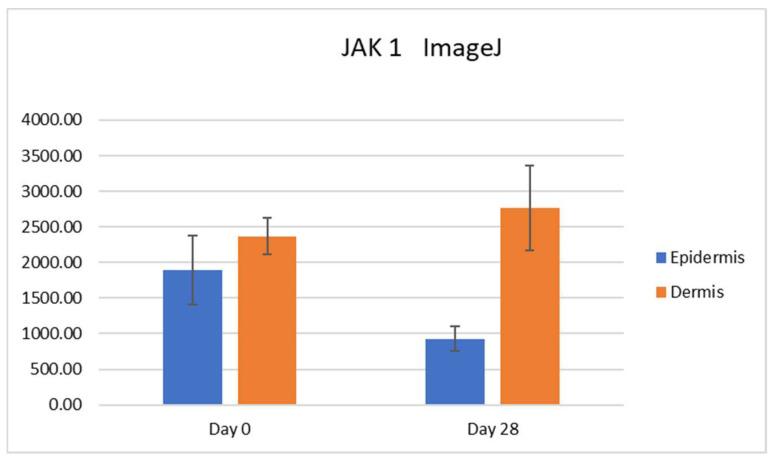
A statistically significant decrease in the number of immunopositive pixels was found on Day 28 compared to Day 0 in the epidermis (*p* = 0.039).

The epidermis and dermis were traced, and the total pixel count of immunopositive pixels per area was calculated. For JAK1, a statistically significant decrease in the number of immunopositive pixels was found on Day 28 compared to Day 0 in the epidermis (*p* = 0.039, Figure 6). No other significant changes were found.

### 3.3. Correlation with Severity of Dermatitis

**Figure 7:**
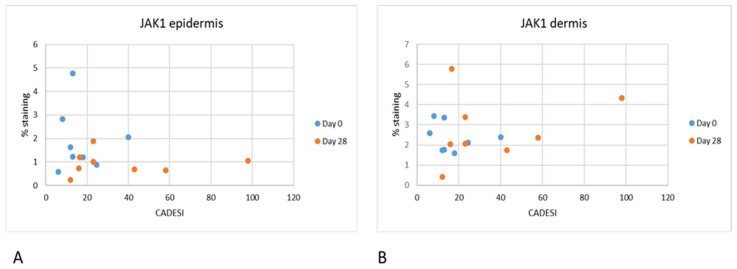
(**A**). This figure shows the correlation between dermatitis scores on the x-axis and % of staining for JAK1, On Day 0 negative correlation R2 is 0.0032; On Day 28 positive correlation R2 is 0.0003. (**B**) on Day 0 negative correlation R2 is 0.0521 and on Day 28 the positive correlation R2 is 0.0739 which is considered weak.

**Figure 8:**
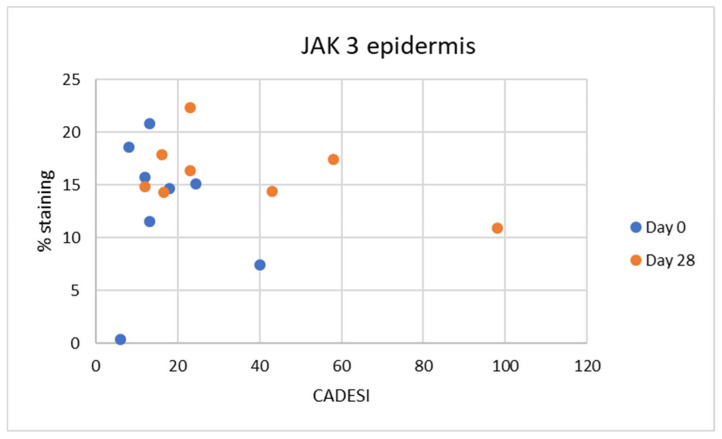
No significant correlations were found between JAK3 staining and severity of dermatitis scores (CADESI). On Day 0 the negative correlation R2 was 0.0151 (Weak), and on Day 28 negative correlation R2 is 0.2707 (moderate).

Correlations were also calculated between the severity of dermatitis (CADESI scores) and semiquantitative measurements of the intensity of scoring for JAK1 and JAK3. No significant correlations were found between the severity of dermatitis and % of positive JAK1 staining (Figure 7) and JAK3 staining (Figure 8).

## 4. Discussion

In our study, we were able to document the expression of JAK1 and JAK3 in canine atopic skin, and we found that there is a decreased expression of JAK1 in the epidermis and increased expression of JAK3 in the epidermis of house dust mite-allergic atopic dogs after allergen exposure. These changes are most likely due to inflammation, but it could also be due to the fact that the mites could have had a proteolytic effect, and it is possible that the results could also be induced by the mites rather than the allergic process. As we did not biopsy normal dogs challenged with dust mites, we are not able to sort out how much of that effect was linked to the allergic inflammation and how much was due to the presence of the mites on the skin. All biopsies used in our studies were taken from atopic dogs and even if the baseline biopsies were from non-lesional skin, atopic skin is always intrinsically different from normal skin.

The decreased JAK1 immunostaining after allergen exposure and flare of clinical signs could be explained by the fact that JAK/STAT signaling is inhibited by SOCS proteins [1,6,13], overexpressed in atopic dermatitis [13]. It is demonstrated in the literature that SOCS directly inhibits JAK1, JAK2, and TYK2, with similar affinity, but has no affinity for JAK3 [16]; this could explain the JAK1/JAK3 imbalance at the end of the experiment, due to SOCS inhibition only on JAK1.

Stains for SOCS proteins were not carried out in our study, and therefore it is unknown if they are overexpressed also in atopic dogs. To the best of the author’s knowledge, the factors modulating the expression of JAK in dogs are not known. This is an important area of research moving forward as modulation of JAKs can play an important role in response to treatment. For example, there are some atopic dogs that simply are non-responsive to treatment with oclacitinib, and the exact reasons for that lack of response are not known. Similarly, there are dogs that are responsive in the early stages of treatment and then stop responding. Correlating the JAK1 expression and the clinical response to oclacitinib could be a very interesting approach to better understand the various clinical outcomes.

In our study, we found JAK1 and JAK3 positive cells in the dermal infiltrate of our biopsies. These cells were identified to be primarily lymphocytes and eosinophils based on Hematoxylin and Eosin staining. In hindsight, it would have been interesting to perform double staining to better characterize which cells express JAK1 versus JAK3 and how that changes during the course of allergen exposure.

It is important to point out that the epidermis gets thicker as a result of allergen challenge and chronic inflammation. We have seen it in this experimental model for canine atopic dermatitis, and it is a common finding in people with atopic dermatitis. This factor needs to be taken into consideration when subjectively evaluating the intensity of immunohistochemistry in biopsy samples. The increased thickness of the epidermis may lead to increased intensity for subjective scores but when the scores are divided by the thickness, this effect is correct. Thus, we feel that the decrease in JAK1 intensity when the thickness factor was taken into consideration is a real finding.

Interestingly in our study, we did not find any correlation between the severity of dermatitis scores and JAK expression. As our study was limited in size, it is possible that significant correlations can be found when examining a larger number of samples. It is also important to point out that the dermatitis scores used in this analysis were the scores for the whole body and not for the specific site that was biopsied. It is possible that with a larger number of samples and focusing on the clinical score of the specific site biopsied a correlation may be found.

As far as immunolocalization is concerned, JAK1 was found in the cytoplasm, and this is the normal localization of JAK family enzymes [6,7]. In fact, after the attachment of the signaling molecule to its transmembrane receptor, activation of the enzymes associated with the cytoplasmic domain of the receptor JAK takes place. When activated, JAKs phosphorylate cytokine receptors, which enables STAT monomers present in the cytoplasm to bind to the complex and form homo- and heterodimers due to tyrosine phosphorylation. Then, activated STATs translocate to the cell nucleus and bind to DNA, enabling the transcription of target genes [6].

In the epidermal layer, JAK3 was overexpressed on Day 28, indicating its crucial role in inflammatory keratinocytes as previously demonstrated [11]. The epidermal expression levels of JAK3 suggest that its inhibition could be a promising approach for topical application [11]. The expression patterns of JAKs have been evaluated in various skin diseases in people, including atopic dermatitis [11]. In particular, phosphorylated and non-phosphorylated forms of JAK/STAT pathway members were analyzed in atopic dermatitis, psoriasis, lichen planus, cutaneous lupus erythematosus, pyoderma gangrenosum, and alopecia areata versus healthy controls. As far as JAK3 and pJAK3 expression is concerned, in the epidermis, it showed a higher expression in all inflammatory diseases except in cutaneous lupus erythematosus. JAK1 and phosphorylated JAK1 were only significantly overexpressed in pyoderma gangrenosum. No significantly different expression was seen for JAK2 and phosphorylated JAK2 in any of the investigated diseases. JAK3 was strongly expressed also in alopecia areata; JAK1 and JAK2 were to a lesser extent also elevated. The resulting downstream cascade seems to be based on the expression levels mainly conducted by phosphorylated STAT2. [11] This is remarkable because alopecia areata is considered to be an IFN-γ driven disease which would result in pan-JAK activation. In this regard, it is important to notice that JAK3 was found to be the only JAK overexpressed in human alopecia areata compared to controls. In veterinary medicine, alopecia areata is also described. An investigation of JAK/STAT pathway members’ expression in this disease would be interesting in order to demonstrate the role of topical or systemic JAK inhibitors in disease regression, as anecdotally reported.

Compared to healthy skin, in the above-mentioned study [11], the expression of both phosphorylated JAK1 and phosphorylated JAK3 was significantly enhanced in the dermal inflammatory cells of most diseases. This overexpression was more pronounced than in the epidermal keratinocytes. None of the inflammatory skin diseases is characterized by an increased expression of JAK2; phosphorylated JAK2 was overexpressed in atopic dermatitis and pyoderma gangrenosum. An interesting finding of the study [11] is that the JAK3 pathway was most upregulated, followed by JAK1 and to a lesser extent JAK2. In the epidermal layer, JAK3 was overexpressed in all diseases (except cutaneous lupus erythematosus), indicating its crucial role in inflammatory keratinocytes. This suggests that selective JAK3 inhibition is the most promising approach for topical application in inflammatory skin diseases, as it can directly reverse the JAK profile of keratinocytes.

The expression of JAKs in the skin is of particular interest as it opens the possibility for topical treatments targeting these pathways. This approach allows a focus on where the suppression is needed, eliminating the need for systemic treatment and the risk of adverse effects. Studies regarding topical JAK inhibitors (tofacitinib, ruxolitinib, brepocitinib) to treat atopic dermatitis in humans are ongoing [17,18]. Ruxolitinib cream has met all primary and secondary endpoints in phase 3 clinical trials for mild-to-moderate atopic dermatitis with minimal treatment-emergent adverse events. Delgocitinib is an approved topical pan-JAK inhibitor for treating atopic dermatitis in adults and children [17]. Based on the structural and functional characteristics of JAKs, as well as specific tissue distribution, JAK3, in particular, has emerged as an ideal target for the treatment of inflammatory or autoimmune diseases [17] as previously demonstrated. To date, no selective JAK3 inhibitor has yet been approved for the treatment of inflammatory and autoimmune diseases [17]. Topical JAK inhibitors are found to be effective and safe in the treatment of atopic dermatitis, and they were observed to significantly improve clinical signs, pain, and pruritus scores compared to placebo in human patients [19].

Moreover, topical JAK inhibitors have a lower risk of adverse effects compared to systemic JAK inhibitors or other immunomodulatory drugs used to treat the disease. The majority of reactions are localized to the application site, and treatment discontinuation is not needed, making this type of treatment appealing. Other topical options for atopic dermatitis are glucocorticoids and calcineurin inhibitors. Topical glucocorticoids have the undesirable adverse effect of cutaneous atrophy, and topical calcineurin inhibitors frequently cause a stinging sensation, at least at the beginning of therapy.

In terms of comparing the efficacy of topical and systemic JAK inhibitors in the treatment of atopic dermatitis, it has been reported that topical forms are significantly more effective than systemic. In particular tofacitinib, ruxolitinib, and delgocitinib had higher achievement in the investigator’s global assessment response compared to topical tacrolimus and phosphodiesterase-4 inhibitors. [17]. Meanwhile, tofacitinib was reported to possess the highest response among the rest of the included medications [17].

JAK3 had the same nuclear staining pattern as reported in previous studies [3,4,10] possibly reflecting nuclear translocation [18]. In a previous study [11], all JAKs and their phosphorylated forms were positive in the epidermis and dermis of inflammatory skin diseases, and they showed a cytoplasmic expression along the cell membrane of keratinocytes, with the exception of pJAK2, which was nuclear. TYK2 and pTYK2 were expressed in the nucleus and in the cytoplasm, with pTYK2 nuclear expression being more pronounced than the cytoplasmatic one. There is evidence that these kinases phosphorylate not only other transcription factors besides the STATs, but that the JAKs function as epigenetic regulators of gene expression. Emerging evidence indicates that the latter role may be of particular significance under physiological and pathological conditions of heightened cellular growth [19]. This was confirmed in a recent publication that reported on JAK3 expression in the nucleus of malignant T cells [20].

Limitations of our study included the small number of dogs and the lack of a normal dog population similarly challenged with the allergen. An additional potential limitation is that an antibody raised against human JAKs was used rather than a specific anti-canine antibody. To the best of the authors’ knowledge, such an antibody is not commercially available. The antibody used is advertised to react to canine JAK, but it is possible that it could bind and cross-react with other proteins. In our study, we did not perform investigations also on phosphorylated forms of JAKs as reported in previous studies [11]. Further studies are needed in order to better determine the phosphorylation of these enzymes and thus their activation or inactivation status.

## 5. Conclusions

In conclusion, we confirmed that both JAK1 and JAK3 are found in canine atopic skin. JAK1 was mostly visible in the dermis while JAK3 was heavily stained in the epidermis besides the dermis. This highlights the critical role of keratinocytes in the initiation and perpetuation of atopic inflammation and suggests that topical inhibitors of JAK3 may be promising when treating inflammatory skin diseases in dogs, like atopic dermatitis. Currently, no topical JAK3 inhibitor is available in both human and veterinary medicine. JAK3 was found to be overexpressed also in human alopecia areata [11]; the investigation of its role in animals should be proven. Since the efficacy of systemic JAK inhibitors in the treatment of dog pruritus is well known [2], the use of topical JAK inhibitors in veterinary dermatology could be investigated. They could be useful in localized forms of pruritus, such as distal limbs, frequently encountered in atopic dogs. The advantage of topical formulations is to deliver a high concentration of drug right where it is needed and minimize the risk of adverse effects that comes with systemic administration of drugs. The limitation of using topical products in veterinary medicine is the presence of hair on the skin and the possibility that the patient may remove the product by licking. In addition, topical products should be applied by the owners. These must be properly instructed about how to apply the therapy and the requirement to use gloves, so cases should be carefully selected. Certainly, the advantage of their use in patients in whom systemic drug administration is contraindicated given concomitant diseases such as cancer is important. In fact, the prevalence of atopic dermatitis has been increasing in recent years, and elderly animals with chronic atopic dermatitis often have comorbidities such as neoplastic, endocrine, or infectious diseases. The use of topical JAK inhibitors can be investigated especially for these patients. Future studies regarding the safety and efficacy of these therapies in veterinary medicine are needed.

## Data Availability

Data will be made available upon reasonable request.
